# Designing a multifaceted telehealth intervention for a rural population using a model for developing complex interventions in nursing

**DOI:** 10.1186/s12912-020-0400-9

**Published:** 2020-02-04

**Authors:** Stephen M. Davis, Amanda Jones, Margaret E. Jaynes, Kori N. Woodrum, Marcus Canaday, Lindsay Allen, Jennifer A. Mallow

**Affiliations:** 1 Department of Health Policy, Management, and Leadership, Robert C. Byrd Health Sciences Center, School of Public Health, West Virginia University, PO Box 9190, Morgantown, WV 26506-9190 USA; 2 Department of Emergency Medicine, West Virginia University, PO Box 9149, Morgantown, WV 26506 USA; 3 Departments of Neurology & Pediatrics, West Virginia University, PO Box 9214, Morgantown, WV 26506 USA; 4 Take Me Home, West Virginia, Bureau for Medical Services, Charleston, WV 25301 USA; 5 Adult Health Department, School of Nursing, West Virginia University, PO Box 9600, Morgantown, WV 26506 USA

**Keywords:** Telehealth, Nursing, Intervention design, Rural, Chronic disease, Medicaid

## Abstract

**Background:**

Telehealth interventions offer an evidenced-based approach to providing cost-effective care, education, and timely communication at a distance. Yet, despite its widespread use, telehealth has not reached full potential, especially in rural areas, due to the complex process of designing and implementing telehealth programs. The objective of this paper is to explore the use of a theory-based approach, the Model for Developing Complex Interventions in Nursing, to design a pilot telehealth intervention program for a rural population with multiple chronic conditions.

**Methods:**

In order to develop a robust, evidenced based intervention that suits the needs of the community, stakeholders, and healthcare agencies involved, a design team comprised of state representatives, telehealth experts, and patient advocates was convened. Each design team meeting was guided by major model constructs (i.e., problem identification, defining the target population and objectives, measurement theory selection, building and planning the intervention protocol). Overarching the process was a review of the literature to ensure that the developed intervention was congruent with evidence-based practice and underlying the entire process was scope of practice considerations.

**Results:**

Ten design team meetings were held over a six-month period. An adaptive pilot intervention targeting home and community-based Medicaid Waiver Program participants in a rural environment with a primary objective of preventing re-institutionalizations was developed and accepted for implementation. To promote intervention effectiveness, asynchronous (i.e., remote patient monitoring) and synchronous (i.e., nursing assessment of pain and mental health and care coordination) telehealth approaches were selected to address the multiple comorbidities of the target population. An economic evaluation plan was developed and included in the pilot program to assess intervention cost efficiency.

**Conclusions:**

The Model for Developing Complex Interventions in Nursing provided a simple, structured process for designing a multifaceted telehealth intervention to minimize re-institutionalization of participants with multiple chronic conditions. This structured process may promote efficient development of other complex telehealth interventions in time and resource constrained settings. This paper provides detailed examples of how the model was operationalized.

## Introduction

The federal government’s Money Follows the Person (MFP) initiative goals include increasing the use of home and community-based services and reducing the use of institutionally based services for people with chronic conditions and disabilities that have transitioned from institutions back into the community [1]. Transitioning individuals from long-term care facilities to their own homes in the community requires multiple key intervention elements including: (a) educating the individual and caregiver about common, unplanned transitions in care (e.g., facility readmission, unintended emergent admission, etc.) and ways to delay or avoid the transition; (b) providing timely communication among everyone involved, including the individual, caregiver and care team; (c) involving the individual and caregiver in establishing goals of care; (d) comprising a strong collaborative interprofessional team; and, (e) implementing evidence-based models of practice [2]. Often, interventions to improve transition outcomes among individuals with chronic conditions in rural areas are ineffective due to poor access to care, inadequate referrals to specialists, and insufficient timeliness of care [3, 4]. Hence, persons living with complex healthcare needs with multiple chronic conditions in rural states require additional unique interventions to maintain the ability to stay in their communities.

Telehealth interventions offer an evidenced based approach to providing patient education, timely communication, goal setting, and linking dispersed healthcare teams [5–9]. Many studies have also demonstrated that telehealth is cost-effective [6]. However, design and implementation of telehealth programs can be complex due to the wide range of devices and applications available and multiple stakeholders who may have competing visions and goals [10, 11]. Additional barriers include assessing a patient’s telehealth needs, technical/legal issues related to sharing protected health information, equipment limitations, inefficient service delivery resources, perceptions of increased staff workload, low staff awareness, and uncertainty regarding remote patient monitoring structures and processes [11]. Addressing these multiple barriers has been associated with successful telehealth implementations. In a review of 45 papers describing telemedicine interventions, Broens et al. [12] observed the following five categorical determinants of success: technology, acceptance, financing, organization, and policy/legislation. Thus, successful implementation in daily practice has been linked to determinants that are important to different stakeholders in different domains [12]. However, the complexity of addressing these multiple determinants has contributed to a gap between the design of evidence-based, pilot telehealth interventions and implementation into practice [12, 13].

The use of theory to structure the design has been suggested as an effective mechanism for ensuring the incorporation of multiple stakeholders’ views with the systemic/environmental context (e.g., technical/legal issues, inefficient service delivery resources, etc.) [13, 14]. Incorporation of a range of stakeholder viewpoints is especially important in the design of telehealth interventions for individuals with long-term conditions (LTCs). The extent to which a telehealth intervention enables relationships between healthcare professionals, peers, and patients and the fit of the designed intervention with a patient’s needs, environment, skills, and capacity have been suggested as important upfront considerations for patients with LTCs [15]. Telehealth intervention for patients with LTCs should also promote self-awareness of vital signs through remote patient monitoring and other technologies [15]. To address these design and implementation concerns, we used a theory-based process that directly incorporates multiple stakeholder views with the environmental context, the Model for Developing Complex Interventions in Nursing (MDCN), to guide the development of a telehealth pilot intervention [3, 16]. The purpose of this article is two-fold: 1) to present a translation of the MDCN into an iterative process that resulted in the design of a telehealth intervention for participants with LTCs; and, 2) to serve as a guide for others wishing to design a telehealth intervention in their own settings and populations.

## Methods

### Setting

The Take Me Home (TMH), West Virginia Program is a federally funded Money Follows the Person (MFP) Rebalancing Demonstration program funded by the Centers for Medicare and Medicaid Services (CMS) [17]. The MFP program supports state Medicaid programs, including the West Virginia Department of Health and Human Resources’ Bureau for Medical Services (BMS), to give older adults and people with disabilities greater choice in where to receive long-term services and supports. The specific goal of this project was to collaborate with, provide insight to, and develop a pilot telehealth demonstration for recipients of Medicaid home and community-based services in the State of West Virginia (WV). WV is ideally suited for this demonstration due to its older population [18] and high disease burden [19]. In 2016, WV had the nation’s lowest healthy life expectancy (63.8 years) [19]. Between 1990 and 2016, WV had the nation’s highest increase (4.4%) in the age-standardized years lived with disability rate [19].

### Participants

As part of this collaboration, a design team comprised of telehealth experts from West Virginia University and state stakeholders affiliated with WVBMS was established. The design team included: a health policy, management, and leadership (HPML) faculty member; an HPML graduate student; a program manager with graduate training in HPML and business administration; two telehealth experts with a combined 30 years experience implementing telehealth in rural communities; and, state stakeholders purposely selected by the TMH Director based on subject matter expertise and familiarity with the target population. State stakeholders included: the TMH Director; the BMS medical director and two BMS nurses; the program manager for the state traumatic brain injury (TBI) waiver and manager of the organization providing care management services to TBI waiver participants; the director of the WV Home and Community-Based Services program; one Medicaid Waiver participant advocate; and, the chief operations officer and regional manager of homemaker services for Medicaid recipients. All decisions related to the telehealth design were made by consensus.

### Theoretical model

**Fig. 1:**
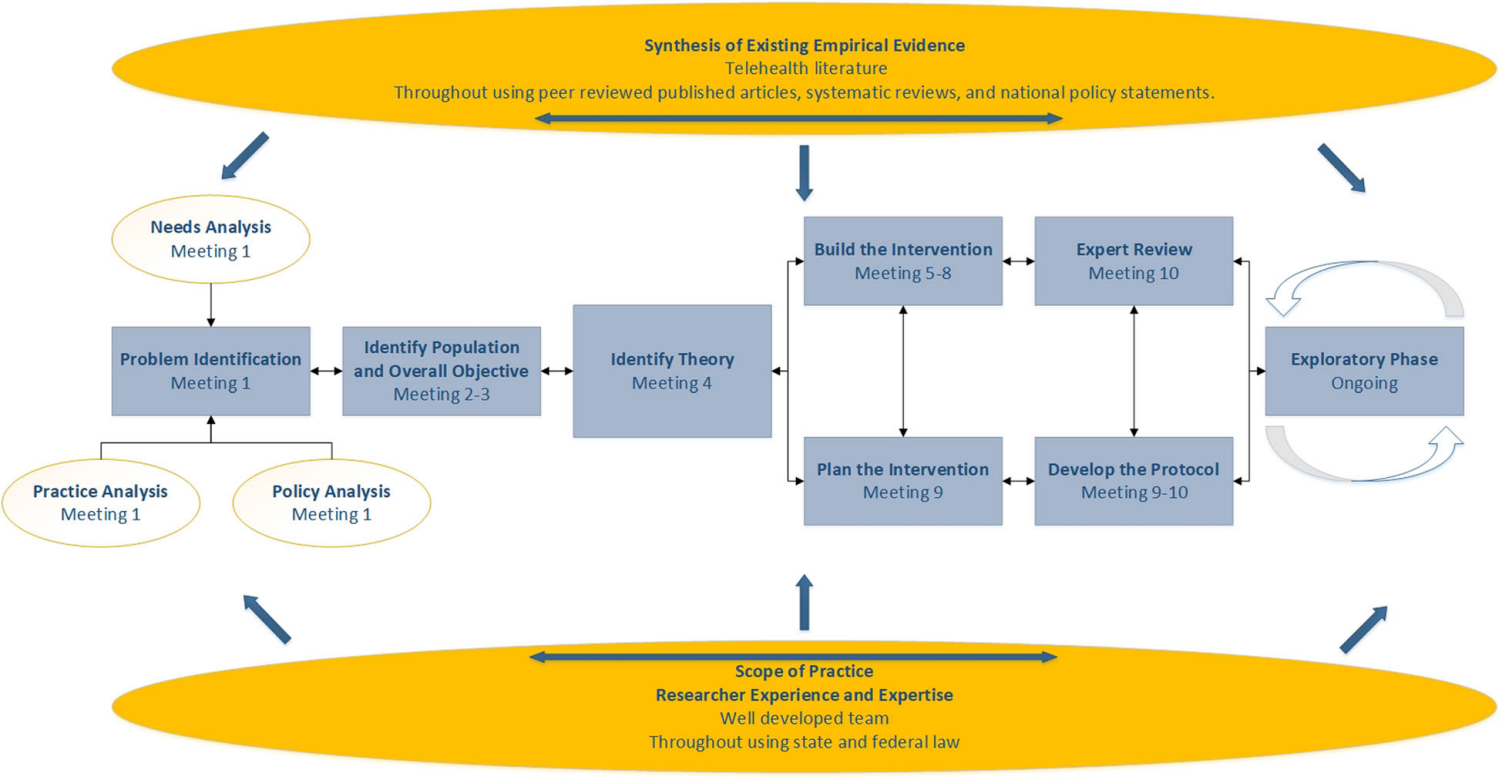
Model for developing complex interventions in nursing

The MDCN (Fig. 1) [3] is an extension of the research model developed by the Medical Research Council to address implementation complexity for interventions with a nursing services component [16]. The first component of the MDCN involves obtaining an in-depth understanding of the problem in need of an intervention. Multiple methods including a review of the existing scientific literature and interviews with key stakeholders can be used to observe the problem and explore contributing factors as well as potential barriers to problem resolution [16]. The second major MDCN component involves solidifying the project scope by defining the intervention’s target population and overall goal and objectives to guide the modeling (i.e., building) process. This component incorporates the results of the literature review and stakeholder meetings from the problem identification process [16]. The third major MDCN component is the selection of a theory to provide structure to the measurement process. Selecting an appropriate theory helps ensure that important variables are included and measured during the intervention design process. Theories specific to nursing interventions are encouraged to account for the complex nature of these interventions that transcend traditional randomized controlled trials [16]. The final major MDCN components involve an iterative process of intervention building and planning, protocol development, and obtaining feedback from potential end-users. This latter part, the expert review, is particularly important in determining the acceptability of the intervention to stakeholders [16]. Engaging potential consumers is one of the nine main strategies that comprise the Expert Recommendations for Implementing Change (ERIC) project [20], and participants have previously been engaged in intervention design to expand telehealth adoption [11].

## Results

Ten meetings over a six-month period were held with the constituted design team. Table 1 summarizes and highlights the salient outputs from each design team meeting guided by the model.

**Table 1:** Meeting agendas and outcomes guided by the model for developing complex interventions in nursing (MDCN)

Meeting	Agenda based on MDCN step	Meeting outcomes
1	Problem identification discussion focusing on needs/practice/policy analyses.	The unique needs and challenges of the population as well as the practice and policy issues were discussed.
2	Discussion and determination of the intervention target population.	Participants with multiple chronic conditions returning to the community from an institution were selected as the target population.
3	Discussion of the intervention and drafting of the overall objectives.	Two primary aims, one focused on cost effectiveness and one focused on project effectiveness, were agreed upon by the design team.
4	Adoption of finalized objectives and selection of the guiding theory.	The Chronic Care Model was selected to inform evaluation of the intervention.
5	Presentation of six possible types of telehealth interventions to the design team.	Based on current scientific evidence, discussions from the group, and a review of Medicaid claims data, a recommendation of a combo of 3 interventions was made.
5&6	Build intervention and protocol development.	Remote monitoring for Chronic Conditions, Remote Nursing Assessment, & Care Coordination telehealth types selected.
7–9	Four potential vendors selected for presentation invitations and final vendor selection.	Each vendor presented available services which were evaluated by the group and a combination of 2 vendors was chosen.
10	Expert review with all stakeholders.	Revisions were made based on feedback.

### Problem identification (meeting 1)

In preparation for this meeting, design team members were asked to think about answers to the following questions:

1. What are the issues with your population? (Needs Analysis)
2. What is it that keeps your population out of the community? (Needs Analysis)
3. What possible telehealth interventions would be beneficial to your population? (Practice Analysis)
4. What are the policy issues? (Policy Analysis)
5. What are the reimbursement issues? (Practice & Policy Analysis)

The design team identified the following as primary concerns: lack of support, isolation, transportation, remoteness, as well as complex inter-personal issues. These issues were further classified according to the Social Determinants of Health [21]:

1. Family & Social Support (e.g., culture of fatalism and self-reliance, access to technology including broadband and hardware such as computers and smart phones, caretaker health, lack of family support, etc.)
2. Education (i.e., health literacy)
3. Income (i.e., poverty)
4. Employment (i.e., lack of quality jobs)
5. Health Behaviors (i.e., activities of daily living deficits)
6. Community Safety (i.e., neighborhood characteristics)

These issues were being challenged through personnel limitations from program policies and provider reimbursement. West Virginia Medicaid only reimburses for real time telehealth communications (i.e., live video). No reimbursement is currently made for store-and-forward or remote patient monitoring [22].

### Identify population and overall objective (meetings 2–3)

The second meeting defined the target population in preparation to identify the overall intervention objectives. The design team selected the following target population: Medicaid Traumatic Brain Injury (TBIW) and Aged and Disabled (ADW) Waiver participants (ages 18 and older) who elect to participate in accessing the Waiver TMH Transition Program to support their transition from long-term care facilities to the community with rolling enrollment to begin in the spring of 2020. As guided by the model, the target population was selected based on the first step, problem identification. To come to this conclusion, the group considered and discussed the information and knowledge gathered through the practice, policy, and needs analysis in relation to the four groups of individuals that qualify for home services already being provided. Based on this knowledge, the group decided that two groups would benefit from telehealth services. Additionally, the group discussion highlighted that if the project was feasible, acceptable, and successful, scalability of the intervention to other populations could happen in the future.

At the third design team meeting, the overall objectives for the proposed pilot intervention were established (Table 2).

**Table 2:** Main telehealth project objectives

Aim 1: Demonstrate cost effectiveness of using telehealth services.	
Sub-aim 1A: Decrease re-institutionalization of TBIW and ADW participants	
Sub-aim 1B: Decrease the number of emergency department and urgent care visits	
Sub-aim 1C: Decrease the number of hospitalizations	
Sub-aim 1D: Evaluate the amount of telehealth services utilized	
Aim 2: Increase quality and safety of home and community-based services through the use of telehealth services.	
Sub-aim 2A: Evaluate participant and provider satisfaction with using telehealth services	
Sub-aim 2B: Increase care coordination for physical and mental chronic illness care using telehealth services	
Sub-aim 2C: Increase access to primary care providers through the use of telehealth services	

*TBIW* Traumatic Brain Injury Waiver; *ADW* Aged and Disabled Waiver

### Identify theory (meeting 4)

At the start of the fourth design team meeting, the university-based team members presented the importance of selecting an academic model to follow. After considering multiple recommendations from the telehealth literature, the group selected the Chronic Care Model [23]. Choosing and following a theoretical model provides structure for how to explain the interactions between the intervention (telehealth) and our measures of success. The Chronic Care Model was specifically chosen after a brief analysis of the concepts in the model for fit to a potential telehealth intervention in this population. The model has previously been used in clinical practice and is designed to improve patient outcomes by changing delivery of care patterns [23].

**Fig. 2:**
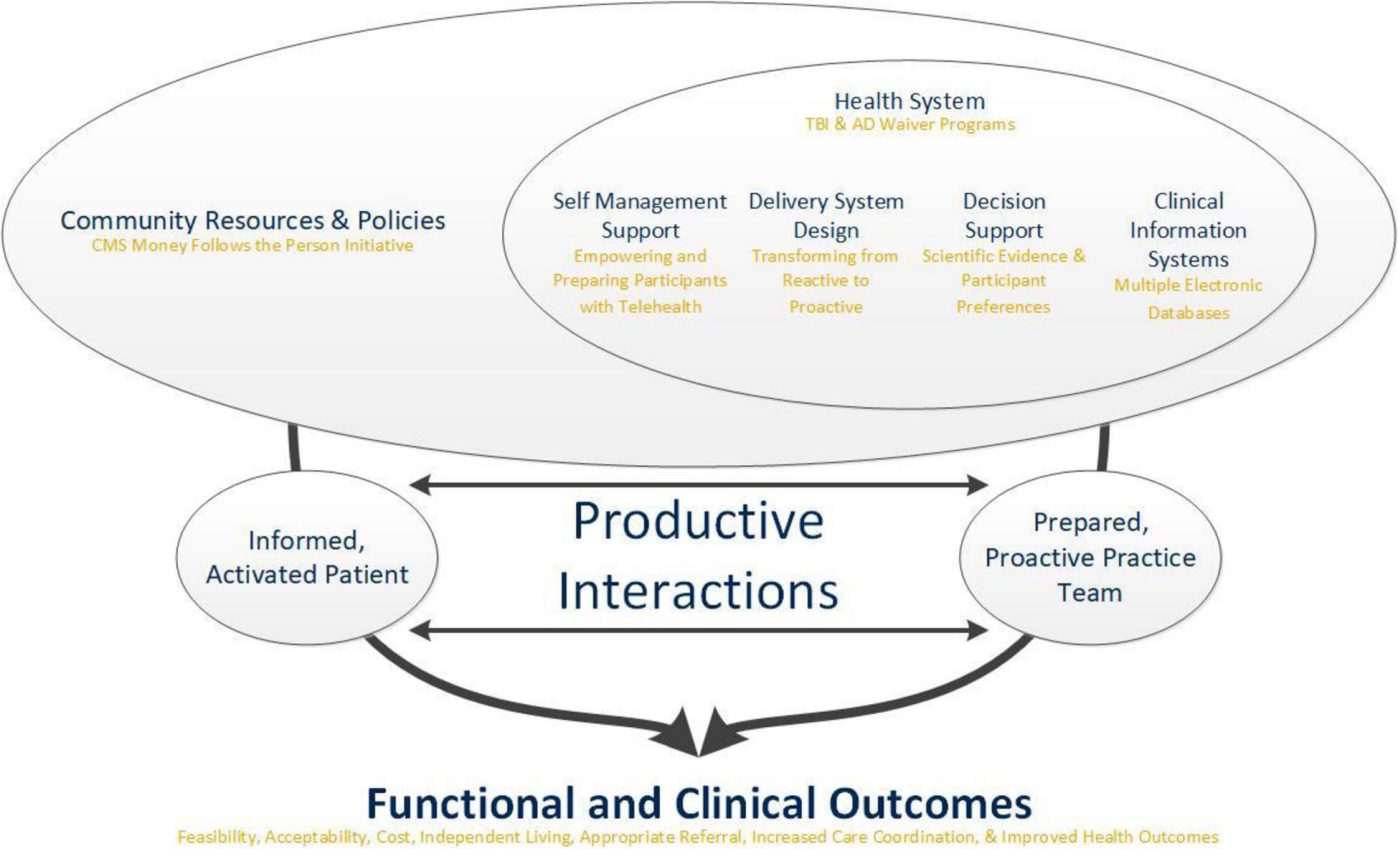
Operationalized chronic care model

Figure 2 is a visual representation of the Chronic Care Model. Each concept in the model has been operationalized for use in this project. The *Community Resources and Policies*, in this case, is the CMS Money Follows the Person initiative. The *Health Systems* of interest are the TBIW and ADW programs. *Self-Management Support* is about empowering and preparing participants to manage their health and healthcare. Interventions that support this approach include telehealth assessment, goal-setting, planning, problem-solving, and follow-up. *Delivery System Design* is transforming a system that is essentially reactive - responding mainly when a person is sick - to one that is proactive and focused on keeping a person as healthy as possible in the community (i.e., making sure that participants get care using structured, planned interactions, like telehealth). *Decision Support* in the model is about promoting clinical care that is consistent with scientific evidence and participant preferences. In this project, the proposed intervention is supported by evidence and is designed to promote patient acceptance. *Clinical Information Systems* organize participant data to measure efficient and effective care. Guided by this model, our designed telehealth interventions include providing timely reminders, identifying when proactive care is needed, individual participant care planning, and sharing clinical information with participants and their healthcare providers to coordinate care. The model depicts productive interactions between an informed participant of the community and the healthcare team that then leads to improved functional and clinical outcomes.

### Build/plan intervention (meetings 5–9)

During the fifth and sixth design team meetings, telehealth medicine experts from the university presented and discussed six main types of telehealth interventions [5]. Based upon this discussion and a review of Medicaid claims data to denote the primary reasons for re-institutionalization of the pilot’s target population (i.e., hospitalizations and emergency department visits; Table 3), three specific proposed telehealth interventions including asynchronous (i.e., remote monitoring) and synchronous (i.e., nursing assessment via telephone and care coordination) approaches were recommended (Table 4).

**Table 3:** Primary reasons for hospitalization and emergency department (ED) visits

ADW service recipients	TBIW service recipients
Hospitalizations Categories (in occurrence order)	Hospitalizations Categories (in occurrence order)
1. Chronic illness exacerbation	1. Chronic Illness exacerbation
2. Infection	2. Infection
3. Pain	3. Mental Health/Substance issue
4. Chest Pain/MI/Afib/Angina	4. Pain
5. Stroke	5. Injury
ED Categories (in occurrence order)	ED Categories (in occurrence order)
1. Chronic Illness exacerbation	1. Infection
2. Infection	2. Mental Health/Substance issue
3. Pain	3. Chronic Illness exacerbation
4. Chest Pain/MI/Afib/Angina	4. Pain
5. Constipation/Nausea/Diarrhea	5. Other Misc.

*TBIW* Traumatic Brain Injury Waiver, *ADW* Aged and Disabled Waiver

**Table 4:** Proposed telehealth interventions

1. Remote Monitoring for Chronic Conditions and Prevention/Early Identification of Infections	
a. Pulse Oximetry with Heart Rate	
b. Blood Pressure Monitor	
c. Glucose Monitor	
d. Weight	
e. Temperature	
f. Fall monitor	
2. Remote Nursing Assessment & Treatment via Telephone	
a. Pain	
i. Assess location, severity (0–10 scale), acute/chronic, aggravating/alleviating factors.	
ii. Plan interventions that include provision of non-pharmacological modalities to treat pain.	
iii. Provide instructions and education on how to treat pain with OTC medications including safe use related to co-morbid conditions.	
iv. Provide instructions on how, when, and where to seek appropriate further care when needed.	
v. Medication Reconciliation	
b. Mental status assessment every 2 weeks	
i. Assess mental health using patient stress questionnaires (PHQ-9, GAD-7, PC-PTSD, and AUDIT).	
ii. Provide appropriate referral and assist with access to care for new or worsening mental health issues.	
c. General assessment of new and on-going issues	
d. Identify patterns and issues with ongoing remote monitoring of chronic conditions and follow-up on missing remote monitoring results for more than 3 days	
3. Care Coordination	
a. Arrange care for new or on-going issues	
b. Medication assessment (i.e., pharmacy pre-wrapped meds) based on medication reconciliation	
c. Follow up on issues after ED/Urgent/PCP visits	
d. Assessment of educational needs related to adherence to self-care behaviors and medications	
i. Plan and provide needed education.	
ii. Evaluate behavior change related to education and need for continued education/intervention.	

*OTC* over the counter, *ED* emergency department, *PCP* primary care provider, *PHQ* Patient Health Questionnaire, *GAD* Generalized Anxiety Disorder, *PC-PTSD* Primary Care Posttraumatic Stress Disorder, *AUDIT* Alcohol Use Disorders Identification Test

Thirteen telehealth vendors that met quality assurance standards including providing safe and effective services direct to patients and meeting the highest standards of care were investigated and considered as potential collaborators for the project. Each vendor was evaluated based on types of services provided, ease of use, years of providing telehealth, cost of services, and interoperability. The top four vendors gave product and service demonstrations to the design team, after which the design team selected two appropriate vendors.

### Develop the protocol (meetings 9–10)

The final two design team meetings involved finalizing the intervention specifics and assessing the feasibility with end users. Vendor contracting and intervention implementation will take 8 weeks and is subject to revisions as needed. While currently residing in a long-term care facility, after being identified as meeting eligibility for either TBIW or ADW services, participants 18 years of age or older who agree to in-home monitoring using technology will be enrolled in the pilot telehealth intervention. Consent to participate in the monitoring as well as evaluation of the intervention will be obtained by the transition coordinator. Once consent has been obtained, assessment of the individual as well as planning for the appropriate telehealth intervention will be performed by the TMH Transition Coordinator using the results of the required assessments with assistance from the project nurse and telehealth vendor clinical staff. The project manager will enter the appropriate variables of measurement into the evaluation database and notify the vendor of the enrolling participant needs. The vendor will deliver the appropriate equipment to the participant’s home and provide instructions for use. Participant information from the remote devices will be sent to the Registered Nurse or Case Manager of the waiver program or primary care provider as necessary per protocol for appropriate coordination and planning of care. Care will continue in this manner for 6 months at which time the vendor will collect the equipment and distribute the evaluation data to the project manager. The project manager will also contact the participant and providers to collect information on satisfaction with the telehealth services.

As telehealth interventions are identified, data on the costs associated with their implementation will be collected by the project manager. Team members will help devise cost data collection protocols to capture outlays, which are expected to include the vendor contract that includes all remote monitoring devices, broadband coverage, and personnel time. These numbers will be incorporated into a summary of start-up (implementation) costs of the interventions. We will also conduct an outcomes analysis, examining changes in rates of emergency department visits, urgent care center visits, and hospitalizations. These outcomes – and their associated costs – will be captured using Medicaid claims data. We will further design a sustainability model that will calculate a capitated per member per month estimate, and will estimate required reimbursement rates for the intervention program to be viable in the long run. Cost-effectiveness will be evaluated in terms of both dollar units and natural units.

### Expert review (meeting 10)

The design team held an expert review with academic experts, medical directors, program directors, managers of patient support services, nurses, telehealth experts, vendors, service providers, participant advocates, potential participants, and students. After a summary of the work accomplished at the 9 meetings with the design team guided by the Model for Developing Complex Interventions in Nursing followed by a “day in the life” case study presentation of the proposed pilot telehealth demonstration, the group was asked whether or not there were any “hard stops”. More specifically, the group was asked whether or not any aspect of the proposed telehealth demonstration project would be unfeasible or logistically untenable in their respective populations. After clarifying the rationale behind the use of a historic control group for the assessment of cost-effectiveness, the use of a landline and/or Wi-Fi, and the potential integration of rehabilitation services in the future, it was determined that there were no aspects of the proposal that were deemed to be unfeasible and in need of redesign. Additional suggestions and comments related to the work were solicited.

## Discussion

To our knowledge, this article is the first to translate the MDCN into a step-wise iterative process. We have described progressing through the process by means of 10 meetings with a group of stakeholders to design a telehealth intervention for a specific population. The use of the MDCN was essential to the successful development of a robust, adaptive, and empirically grounded telehealth intervention. Telehealth interventions have been developed and used in other states to improve care access and outcomes [7]. A structured process intended to meet the unique needs of the population in West Virginia has guided the development of this pilot intervention. The telehealth intervention will be made available to individuals transitioning from institutional care to their communities. Combining multiple health sensors, education, reminders and access to health-care providers diminishes the burden individuals face as they are managing multiple chronic conditions while adjusting to independent life in the community. This combination of services is intended to decrease care complexity for both the individual patients, their communities, and healthcare service providers. Feasibility and acceptability for both patients and healthcare providers will be evaluated. In addition, efficacy and cost of the intervention will be evaluated and will be used to inform decisions about the sustainability and scalability of a larger intervention. While the first implementation of this telehealth is targeted to a specific population, future use is intended for other populations and practice settings if the results of this trial are successful. The content of this intervention in its present iteration is reflective of current empirical evidence about the use of telehealth, a specific needs analysis, and has been adapted based on feedback from a wide variety of stakeholders. This translated process could be adapted to design other interventions in a variety of settings and clinical populations. For example, health systems could use this approach to implement programs in response to a community needs assessment.

There are limitations to this type of approach. Assembling the right stakeholders requires connecting dispersed individuals with academic and pragmatic knowledge. Additionally, the ability to engage in open collegial, and multi-directional dialogue is required. Time can be an issue in working through the process in multiple ways. Scheduling meetings requires gathering multiple busy individuals and in addition to the time required in meetings, independent work and reflection is required between meetings. Lastly, the academic research process of designing large randomized controlled trials may not be possible within the given workflows and cost constraints of established practices. Control groups, population focus, and length of intervention may be based on cost of implementation instead of the gap in the current science.

## Conclusions

A process driven approach facilitated the development of a pilot telehealth demonstration intervention. This demonstration, funded by West Virginia’s Money Follows the Person Program, will provide critical information to promote scalability of telehealth services to other home and community-based services within West Virginia.

## Data Availability

Data sharing is not applicable to this article as no datasets were generated or analysed during the current study.

## Abbreviations

ADW: Aged and Disabled Waiver
BMS: Bureau for Medical Services
CMS: Centers for Medicare & Medicaid Services
HPML: Health Policy, Management, and Leadership
LTC: Long-Term Conditions
MDCN: Model for Developing Complex Interventions in Nursing
MFP: Money Follows the Person
TBI: Traumatic Brain Injury
TBIW: Traumatic Brain Injury Waiver
TMH: Take Me Home
WV: West Virginia
